# Risk of work-related violence in England and Wales

**DOI:** 10.1093/occmed/kqab145

**Published:** 2021-10-11

**Authors:** J A Edwards, P Buckley

**Affiliations:** People Services, The Open University, Wilson Building, Walton Hall, Milton Keynes MK7 6AL, UK; Statistics Branch, Statistics and Analytics, Science Directorate, Health and Safety Executive, Redgrave Court, Bootle, Liverpool L20 7HS, UK

**Keywords:** Crime Survey of England and Wales (CSEW), work-related violence (WRV), work-related violence assault (WRVA)

## Abstract

**Background:**

There is an urgent need to address high levels of work-related violence (WRV) in Britain to improve the workplace experience of both employers and employees.

**Aims:**

This study specifically explores prevalence rates of work-related violence assaults (WRVAs) for staff at high risk that have supervisor responsibilities and work full-time.

**Methods:**

Five years of data were used for analysis from the Crime Survey of England and Wales (CSEW). Data were filtered for victims of crime from a working population in Britain.

**Results:**

Forty-four per cent of respondents who are supervisors and have full-time jobs report that they had been victims of WRVAs. This figure is higher than other previous studies of WRV.

**Conclusions:**

The current study provides further alternative evidence to support high prevalence rates of WRV by selecting for analysis specific work characteristics data that are strongly associated with WRV (supervisor responsibilities and full-time work). It is therefore recommended that future work explores different means of intervention for organizations to implement within the workplace to reduce WRV and protect workers from harm.

**Key learning points**

**What is already known about this subject:**

- It has been established that work-related violence in the UK is on the increase, which has a widespread negative effect upon both employers and employees.
- Work-related violence is an under-researched field of work.
- This study needed to be done to directly explore prevalence rates of work-related violence for the most vulnerable group of workers, full-time supervisors.

**What this study adds:**

- This study further contributes to the work-related violence literature by providing robust longitudinal evidence to support the need for interventions to reduce work-related violence.
- The key message from this study is to draw attention to the most at-risk group of workers of work-related violence assaults which are supervisors with full-time jobs.

**What impact this may have on practice or policy:**

- The legal implication of this study highlights the need for employers to protect employees from harm at work.
- The impact of this research is to supply employers, policymakers and legislators with information and guidance on how to design interventions, while focusing primarily on the most vulnerable group of employees (supervisors/managers and full-time workers).

## Introduction

It has been established that work-related violence (WRV) in the UK is on the increase, which has a widespread negative effect upon both employers and employees [1,2]. Violence at work can adversely influence staff psychological well-being (stress, injury and satisfaction) as well as organizational performance (reputation, legal enforcement, sick pay and turnover). The Crime Survey of England and Wales (CSEW) in 2017/18 reported an 8% increase in WRV compared to the previous year. Assaults are more frequent than threats. Data from a sample of victims of crime within the working population show that 31% of respondents reported that they had been assaulted in the workplace, compared to 22% of threats at work [3]. Two of the strongest characteristic predictors of WRV are working hours and managerial/supervisory job roles [1,2].

The aim of the current study was to look at the variables/characteristics of highest prevalence and interest in relation to WRV based on the results from previous research [1,2]. This will allow the most at-risk group of victims of WRV to undergo a detailed analysis to explore prevalence rates (specifically assaults). Vulnerable groups of employees can be identified and plans for training interventions can be implemented within the workplace to manage and reduce incidents of WRV.

## Methods

**Figure 1.**
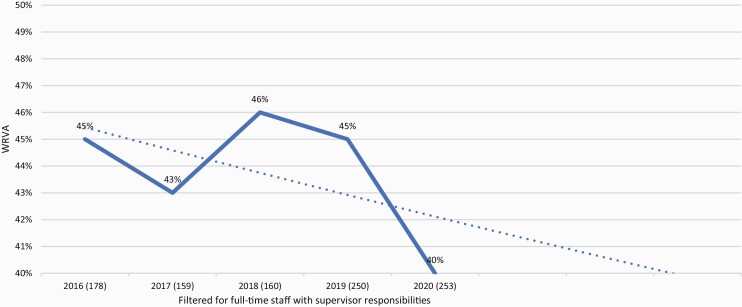
WRVAs in England and Wales from 2016 to 2020. Filtered for full-time staff with supervisor responsibilities.

Data from the CSEW is used from years 2015/16–2019/20 to provide consistency of results. A representative sample of households in England and Wales was used via the Postal Address File (PAF). Home Office data from the CSEW was filtered for WRV variables via the Health and Safety Laboratories (HSL) and the Office for National Statistics (ONS). Data were filtered based on the analytic procedure proposed by Jones *et al*. [4]. Victims of crime within the working population provide the five samples of data from questionnaire-based interviews collated within the UK. Ethical approval was granted by the Home Office and the ONS. Individual number of hits for each year can be seen in Figure 1. Average sample size across the 5 year was 200.

The Statistical Package for the Social Sciences (SPSS) software was used for statistical analysis. Data were cut to support analytical methods via binary responses for ‘Assault at work′ and ‘Assaults not at work′.

## Results

Since there is no specifically designed WRV data collated from the CSEW, data were filtered for victims of crime that had been assaulted at work, had supervisory tasks and worked full-time. Only supervisor and full-time employee data were used for analysis. Simple frequency analytical techniques were used to calculate the prevalence levels across the individual years of data.

Figure 1 shows on average across the 5 years that 44% of workers from the current filtered population report that they have been victims of work-related violent assaults (WRVAs). These findings consistently provide longitudinal evidence that a high percentage of employees who specifically have supervisory and full-time working characteristics, and who have been victims of crime, experience WRVAs.

The exponential trendline shown in Figure 1 reflects the pattern of prevalence for WRVAs across the years. The highest percentage of reported WRVAs was in 2018 at 46%.

## Discussion

The current study shows on average that 44% of employees who work full-time and have supervisory responsibilities report incidents of assault at work.

This contributes to research in the field by providing prevalence rates specifically for WRVAs, rather than simply WRV, which has not previously been reported within the literature. These findings filtered for supervisor provision and full-time employees produce a greater level of violence at work compared to other studies [1–3,5]. Using the present study′s focused analytic approach, prevalence rates of WRV are approximately double what they are for other similar studies provided by authors in the field [1,2].

The dip in WRVAs in 2019/20 shown in Figure 1 is most probably due to the imminent COVID-19 pandemic. Staff for most of 2020 have worked from home where applicable and/or have implemented various flexible working patterns to restrict contamination of the virus. If the 2020 data were consistent with the averaged results from 2015/16–2018/19, the overall average prevalence rate across 5 years would be even higher at 45%. Apart from 2020, the results across 4 years from 2015/16–2018/19 shown in Figure 1 are relatively stable, indicating there is a consistent pattern of high rates of WRVAs in the UK.

Interpretation of the results suggests that the hierarchical relationship between supervisors and their respective employees is more precarious than other working relationships. Working full-time, as opposed to part-time/fractional-time, also exposes the potential victim of WRVAs to a greater number of hours at work.

The present results highlight the essential need to protect principally vulnerable groups of staff from WRV, and in particular WRVAs. This short paper has been purposely produced to be brief, concise and to the point, as it is a part revision of a previous published paper [1,2]. What′s been added to this paper is a more focused approach, through filtering specifically for a sample of employees who have supervisory commitments and work full-time hours, where figures have been produced to reflect the extent of the problem associated with WRV in the UK.

The overall utility of this study is to focus attention on the important need to reduce the impact of WRVAs in the UK, and to provide data to support evidence-based interventions. For example, a standardized toolkit could be designed and implemented within UK workplaces to help decrease levels of WRV, similar in principle to the HSE Management Standards for work-related stress Indicator Tool.

Furthermore, the Health and Safety at Work act (1974) states that there is a legal obligation for employers to protect employees from hazards at work such at WRV [6]. There is an important need to introduce interventions within the workplace to combat the risk of both WRV in general and WRVAs. By doing so, this will provide mechanisms and procedures for clinicians, policymakers and employers to follow in order to reduce WRV.

## Funding

This work was supported by the Health and Safety Executive (PRJ1228).

## Competing interests

None declared.
